# CD38 and CD58: A Gallant Savior in Under-Resourced Laboratories

**DOI:** 10.7759/cureus.72968

**Published:** 2024-11-04

**Authors:** Aditi Agarwal, Deepshikha Rana, Shilpi More, Meera Sikka, Pooja Dewan, Mrinalini Kotru

**Affiliations:** 1 Pathology, University College of Medical Sciences, New Delhi, IND; 2 Pediatrics, University College of Medical Sciences, New Delhi, IND

**Keywords:** acute lymphoblastic leukemia, b-cell acute lymphoblastic leukemia, flowcytometry, mrd lite, pediatrics, screening

## Abstract

Background

B-cell acute lymphoblastic leukemia (B-ALL) is one of the most common childhood malignancies and comprises almost the majority of acute leukemias in children. At the end of induction, minimal residual disease (MRD) is one of the most important prognostic indicators. MRD is commonly detected by multiparametric flow cytometry. Interpreting panels consisting of multiple tubes requires a lot of expertise and is subject to stringent standardization processes. Hence, there arises a need for an easier alternative that can be more universally applicable and reduce complexity in interpretation, resulting in uniform reporting and cost-effectiveness, especially in resource-deficient settings, without compromising clinical outcomes. Therefore, the aim is to find the usefulness of the MRD “lite” panel for post-induction MRD detection in pediatric B-ALL using CD38, CD10, CD34, CD19, and CD45 compared to the “standard tube” panel.

Methods

The study included 25 children diagnosed with B-ALL and undergoing treatment. MRD detection was performed on day 28 of post-induction chemotherapy using a standard tube panel consisting of three tubes (gold standard) and our single tube MRD “lite” panel. The results obtained by the two were compared.

Results

The sensitivity and specificity of the MRD “lite” panel were calculated as 100% and 84.2%, respectively, taking the standard tube panel as the gold standard. CD10, CD20, and CD200 were valuable in distinguishing MRD-positive cases from MRD-negative cases. Moreover, the MRD “lite” panel had 100% concordance between the two observers, suggesting simplicity in assessment and, hence, wider applicability.

## Introduction

Minimal residual disease (MRD) is the persistent presence of recalcitrant blasts in the bone marrow even after receiving chemotherapy at a level that cannot be detected by the usual morphological techniques. It is one of the most important prognostic factors for acute lymphoblastic leukemia (ALL) apart from other factors such as age, total leukocyte count (TLC), and cytogenetics [1]. Residual blasts can be detected with a sensitivity of up to 0.01% by multiparametric flow cytometry (MFC) [2]. Early MRD detection is useful in leukemias sensitive to therapy that are likely to become MRD negative after one to two weeks of chemotherapy, and a reduction in therapy during the intensification phase of treatment can be done. MRD can be detected up to the order of one per 10,000 events using multiple tubes with various markers in different combinations [3]. However, as more markers are being introduced, the technical complexity and the cost have also increased proportionately. Thus, there arises a need to design a panel that decreases this complexity in interpretation and minimizes the cost to realize its full diagnostic potential.

Currently, MRD is performed at higher centers using larger and variable panels with different cocktails of antibodies that are not standardized. Campana et al. and Chatterjee et al. from India conducted studies on MRD detection by MFC using multiple marker combinations and found its prognostic significance remarkable [2,4]. They concluded that post-treatment detection of MRD is of utmost importance and should not be omitted, although the they panels used were complex and not standardized. Increased usage of complex panels may or may not significantly increase the test performance. Hence, there is an utmost need to devise an optimal testing panel in terms of minimizing cost and difficulty while maximizing clinical performance. Our present study attempts to test for the sensitivity of the MRD “lite” panel using CD38, CD10, CD19, CD34, and CD45 for the diagnosis of residual disease in B-cell acute lymphoblastic leukemia (B-ALL).

This article was previously presented as a meeting abstract at the 30th Annual Conference of Delhi Society Haematology on September 15, 2019, titled “An Algorithmic Approach to B-All MRD Using Flow Cytometry.”

## Materials and methods

This cross-sectional study was conducted in a tertiary care hospital in New Delhi, India. Thirty-one pediatric patients with B-ALL undergoing treatment were enrolled in the study. However, 25 cases who completed induction chemotherapy as per institutional protocol were studied for MRD status. The remaining six were lost to follow-up. Approval from the Institutional Ethics Committee for Human Research(IEC-HR/2017/32/104) was obtained.

A complete blood count (CBC) was performed on a Mindray 6200 (Shenzhen Mindray Bio-Medical Electronics Co., Ltd, Shenzhen, China), along with peripheral smears, bone marrow aspirate smears, and flow cytometry, for diagnosis and baseline immunophenotyping on day 0 and for MRD detection upon completion of induction chemotherapy.

Immunophenotyping was performed using a five-color flow cytometer (FC-500, Beckman Coulter, Brea, CA). The sample was obtained in K-EDTA, and the standard lyse/wash technique was used. The samples were stained with cMPO (cytoplasmic myeloperoxidase), cCD79a, cCD3, CD19, CD10, CD20, CD13, CD33, CD64, CD117, Tdt, HLA-DR, and CD1a for diagnosis (Table 1).

**Table 1:** Fluorochrome-labeled antibodies used and their clones used in our study

Antibody	Fluorochrome	Clone
Anti-CD19	R phycoerythrin cyanin 5.1 (PC.5)	Beckman Coulter clone-J3-119
Anti-CD34	R phycoerythrin cyanin 7 (PC.7)	Beckman Coulter clone-581
Anti-CD45	R phycoerythrin-Texas red-X (ECD)	Beckman Coulter clone-J33
Anti-CD20	Fluorescein isothiocyanate	Beckman Coulter clone-B9E9 (HRC20)
Anti-CD10	Phycoerythrin	Beckman Coulter clone-ALB1
Anti-CD58	Fluorescein isothiocyanate	Beckman Coulter clone-AICD58
Anti-CD200	Phycoerythrin	Beckman Coulter clone-OX-104
Anti-CD38	Fluorescein isothiocyanate	Beckman Coulter clone-T16

Also, an MRD panel (Table 2) was put at the time of diagnosis wherever the sample was sufficient.

**Table 2:** The standard panel and MRD “lite” panel put for MRD detection FITC, fluorescein isothiocyanate; MRD, minimal residual disease; PE, phycoerythrin

		FITC	PE	ECD	PC.5	PC.7	
Standard panel	Tube 1	CD20 10 μL	CD10 10 μL	CD45 10 μL	CD19 10 μL	CD34 5 μL	45 μL
	Tube 2	CD58 20 μL	CD10 10 μL	CD45 10 μL	CD19 10 μL	CD34 5 μL	55 μL
	Tube 3	CD38 10 μL	CD200 5 μL	CD45 10 μL	CD19 10 μL	CD34 5 μL	40 μL (test)
MRD “lite” panel		CD38 10 μL	CD10 10 μL	CD45 10 μL	CD19 10 μL	CD34 5 μL	45 μL

Bone marrow aspirates of all patients were tested for MRD using the standard three-tube panel along with the MRD “lite” panel (Table 2) at the end of induction chemotherapy. The fluorochrome-labeled antibodies and their clones used in our study are mentioned in Table 1.

Sample acquisition

Flow cytometry was performed on the Beckman Coulter FC-500 flow cytometer using Cytometry List Mode Data (LMD) Acquisition & Analysis Software (CXP Analysis 2.2). The samples were acquired for maximum events up to 600 seconds, or a total of 12,00,000 events, whichever was first. Analysis was performed on the list mode files in the CXP Analysis 2.2 software.

Calculation of MRD percentage positivity

The standard tube panel was used as a gold standard for the diagnosis of MRD. MRD percentage was calculated in each tube individually. The highest MRD percentage of the three tubes was taken as the MRD percentage of the panel.

Blast events

MRD positivity = (blast events / total mononuclear events) x 100

Total mononuclear events

For the assessment of MRD on the MRD “Lite” panel, two independent observers reported MRD individually. They were blinded to the clinical findings, CBC, bone marrow remission status, and MRD status by the three-tube panel.

Statistical analysis

Statistical analysis was performed using MS Excel (Microsoft, Redmond, WA) and SPSS software (IBM Corp., Armonk, NY). Mean, standard deviation, and median were calculated for all the quantitative parameters in the study. For paired categorical variables such as hemoglobin (Hb), TLC, platelet count (PC), absolute neutrophil count (ANC), absolute lymphocyte count (ALC), and blast percentage at the time of diagnosis and post-induction chemotherapy, the McNemar-Bowker test was used. For continuous variables such as the positivity percentage of all the CD markers, correction with MRD positivity on Levene’s test for equality of variances was used. For the correlation of MRD positivity with all other hematological parameters before and after treatment, logistic regression was used.

## Results

The study included 31 cases, but six cases were lost to follow-up; thus, the remaining 25 pediatric B-ALL cases were studied. The age of the patients ranged from 2 to 18 years, with a mean ± SD of 7.1 ± 4.6 years, including 32% females and 68% males (Table 3).

**Table 3:** Age and sex distribution in B-ALL patients (n=25) B-ALL, B-cell acute lymphoblastic leukemia

	Number	Percentage (%)
Age < 10 years	20	80
Age > 10 years	5	20
Male	8	32
Female	17	68

The clinical profiles of the patients at the time of presentation are summarized in Table 4.

**Table 4:** Clinical profile of pediatric B-ALL patients at the time of diagnosis (n=25) B-ALL, B-cell acute lymphoblastic leukemia

Clinical presentation	Number	Percentage (%)
Weakness and fatigue	24	96
Fever	21	84
Neck swelling	16	64
Bone pains	14	56
Weight loss/loss of appetite	10	40
Gum bleeding or bleeding from any other site or purpura	10	40
Frequent infections	8	32

At the time of diagnosis, Hb ranged from 2.5 to 10.6 g/dL, with a mean ± SD of 7.4 ± 1.4 g/dL, TLC ranged from 4,900 to 54,000 /µL, with a mean ± SD of 25,948 ± 11,314/µL, and PC ranged from 190 to 304 x 10^9^/L, with a mean ± SD of 900.4 ± 604.9 x 10^9^/L. Blast percentage ranged from 25% to 99%, with a mean ± SD of 81 ± 18%. ANC and ALC ranged from 0 to 7,000/mm^3^, with a mean ± SD of 1,840 ± 1,952/mm^3^, and 212 to 9,720/mm^3^, with a mean ± SD of 2,657 ± 2,602/mm^3^, respectively (Table 5).

**Table 5:** The mean ± SD and range of various hematologic parameters in the patients at diagnosis and post-induction (n=25). *A p-value of <0.05 is considered significant Hb, hemoglobin; TLC, total leucocyte count; ANC, absolute neutrophil count; ALC, absolute lymphocyte count

Parameter	At diagnosis		Post-induction		p-Value*
	Mean ± SD	Range	Mean ± SD	Range	
Hb (g/dL)	7.4±1.4	2.5 to 10.6	10±1.4	6.8-12.8	0.002
TLC(/µL)	25,948±11,314	4,900-54,000	4,864±2,222	1,200-8,900	1
Platelet count (x10^9^/L)	900.4±605	190-304	209.4±115	59-533	0.0001
Blast %	81±18	25-99	0	0-4	0.0001
ANC(/mm^3^)	1,840±1952	0-7,000	2,589±1931	490-9,116	0.07
ALC(/mm^3^)	2,657±2602	212-9720	2,607±2015	360-10,600	0.22

During the post-induction phase of chemotherapy, Hb ranged from 6.8 to 12.8 g/dL with a mean ± SD of 10 ± 1.4 g/dL, TLC ranged from 1,200 to 8,900/mL with a mean ± SD of 4,864 ± 2,222/mL, PC ranged from 59 to 533 x 10^9^/L, with a mean ± SD of 209.4 ± 115x10^9^/L, and blast percentage ranged from 0% to 4%. ANC and ALC ranged from 490 to 9,116/mm^3^, with a mean ± SD of 2,589±1931/mm^3^, and 360 to 10,600/mm^3^ with a mean ± SD of 2,607±2015/mm^3^, respectively. The p-values for Hb, PC, and blast percentage before and after treatment were statistically significant (p-value of Hb=0.002, p-value of PC=0.0001, and p-value of PC=0.0001) (Table 5).

Bone marrow aspirate examination showed hypercellularity with near-total to total replacement by blasts. Post induction, all cases showed bone marrow in morphological remission. All the cases of B-ALL were diagnosed based on the positivity of CD19 along with the co-positivity of any two of CD10, CD20, and cCD79a by MFC.

Post-completion of treatment MRD detection by flow cytometry using a standard tube panel and MRD “lite” panel was done (Table 6).

**Table 6:** Mean positivity percentage of all markers in MRD-positive and MRD-negative cases and their respective p-values for the standard panel (gated CD19 positive cells) (n=25). A p-value of <0.05 is considered significant. MRD, minimal residual disease

CD markers	Mean positivity %	Mean positivity in MRD-positive cases	Mean positivity in MRD-negative cases	p-Value
CD34	48.2	57.7±27.5	45±18.6	0.22
CD10	52.3	84.5±13.6	42±23.3	<0.001
CD20	81.7	66.7±23.4	86.4±15.5	0.02
CD58	79.2	87.5±9.6	76.6±16.4	0.06
CD200	83.3	95±4.6	81±22	0.017
CD38	73.5	79.3±33.7	71.6±22	0.52

When comparing the individual markers between MRD-positive and MRD-negative patients, the p-value of CD10 and CD20 was <0.05 (when equal variance was assumed). When equal variance was not assumed, CD200 (p-value=0.017) was statistically significant and CD58 (p-value=0.06) showed a trend toward being different between the two groups. MRD was found to be positive in 24% (6/25) cases, with the cut-off for positivity taken as >0.01% of mononuclear events. In our study, three out of six cases, despite being positive for cALLA (common acute lymphoblastic leukemia antigen), were MRD-positive. The percentage of MRD positivity ranged from 0.5% to 2.14%. Cluster formation was seen in all MRD-positive cases in one or more tubes. The MRD-positive blasts formed a distinct population from the hematogones in all positive cases except in one case, where two distinct clones of blasts were seen as two separate clusters, one of which was intermixed with the hematogones.

Age, sex, Hb, TLC, ANC, ALC, and PC before and after treatment were further correlated with MRD positivity. No statistically significant correlation was found between the MRD-positive and MRD-negative cases (p-value of Hb=0.560, p-value of TLC=0.912, p-value of ANC=0.125, p-value of ALC=0.214, and p-value of PC=0.274) (Table 5).

**Figure 1:**
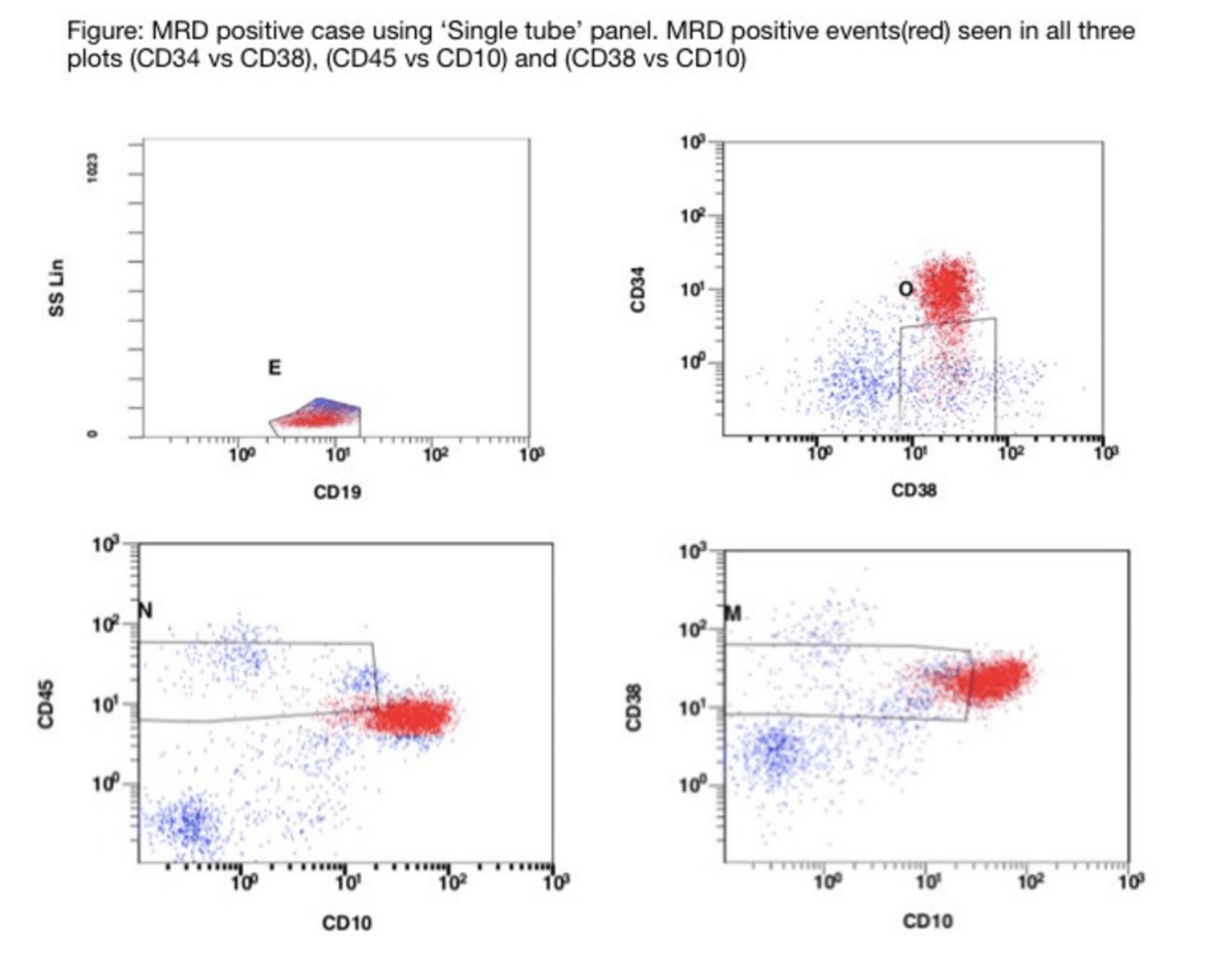
MRD-positive cases using the “single tube” panel. MRD-positive events (red) can be seen in all three plots (CD34 vs. CD38, CD45 vs. CD10, and CD38 vs. CD10). MRD, minimal residual disease

MRD detection by flow cytometry using the MRD “lite” panel showed MRD positivity in 36% (9/25) cases. The percentage of MRD positivity ranged from 0.03% to 64.9%. Positivity was studied in three combinations of markers, which were CD38 vs. CD10, CD45 vs. CD10, and CD38 vs. CD34 (Figure 1).

Two observers analyzed all the cases to rule out observer bias. There was 100% concordance between the two observers. Cluster formation outside the hematogone region in any one of the marker combinations was considered MRD-positive. A comparison of every MRD-positive case for both panels was done (Table 7).

**Table 7:** Comparison of MRD positivity using the three-tube panel versus the single-tube panel (n=9) MRD, minimal residual disease

Case number	Three-tube panel	Single-tube panel
3	1.38	0.4
6	1.2	0.7
9	Negative	1.39
10	Negative	0.03
11	0.9	1.33
12	2.14	1.27
14	1.05	2.8
15	0.5	0.05
23	Negative	64.9
23	6	9

Overall, 64% (16/25) cases were found to be negative using both panels, and 24% (6/25) cases were positive using both panels. Discordance was observed in 12% (3/25) cases, which were positive using the MRD “lite” panel, whereas they were found to be negative using the standard panel. Hence, there were three false-positive cases by the MRD “lite” panel. Hence, the sensitivity and specificity of the MRD “lite” panel were calculated as 100% and 84.2%, respectively, taking the MRD “lite” panel as the gold standard. The difference between the two panels was not statistically significant (p-value=0.25). The negative predictive value was calculated as 100%, while the positive predictive value was 66.67% (Table 8).

**Table 8:** Sensitivity, specificity, and positive and negative predictive values for MRD “lite” panel MRD, minimal residual disease

MRD “lite” panel	
Sensitivity	100
Negative predictive value	100
Positive predictive value	66.67
Specificity	84.2

## Discussion

The detection of submicroscopic levels of blasts in the bone marrow, known as MRD detection, is used as a prognostic tool. It has emerged as one of the major prognostic markers, along with age, TLC, and cytogenetics. MRD is performed on multicolor flow cytometry using multiple markers. Various centers lack standardization and are using an infinite set of markers for detection. Moreover, using so many different antibodies makes it a very expensive investigation. Hence, this study was conducted to find the utility of the limited five markers as the MRD “lite” panel in detecting MRD compared to the standard panel, which had 15 markers in three tubes. In our study, we included 25 children who presented to the OPD or the emergency with symptoms of leukemia and were later diagnosed with B-ALL using MFC and then followed up through therapy. On day 28 post-induction, we performed MRD detection by flow cytometry using a three-tube panel and the MRD “lite” panel. The results of both were compared along with all the other hematological markers.

A complete evaluation of the hematologic profile of all the patients was conducted at the time of diagnosis and also post-completion of the induction phase of chemotherapy, and the p-value was calculated. Hb, PC, and blast percentage had a significant correlation with the treatment outcome. It is known that due to infiltration of marrow by blasts, there is bone marrow failure resulting in thrombocytopenia at presentation. In this study also, 76% of patients presented with thrombocytopenia. These parameters do not have known prognostic significance at the time of diagnosis, although post-induction therapy suggests recovery of these counts [5]. High TLC at the time of diagnosis is considered an adverse prognostic factor, as noted in our study. Post-induction chemotherapy, the mean Hb was 10g/dL, TLC was 4,864/µL, and PC was 209.4 x 109/L, suggesting that, overall, there was a recovery of counts at the end of induction. De Angulo et al. in a study on patients with AML and ALL receiving induction chemotherapy showed that ALC<350/mL on day 15 of induction therapy was significant in predicting the outcome of treatment in ALL. They measured weekly ANC, ALC, and PC to come to this conclusion. It was an independent factor for poor outcome of treatment in ALL [6]. Although de Angulo et al. concluded that ANC, ALC, and PC are associated with poor outcomes, in our study only a trend was seen between ANC and MRD (p-value=0.07), which was not statistically significant. A larger sample size could facilitate reaching similar results.

According to Raetz et al., complete remission (CR) is defined as <5% blasts in the bone marrow with no other evidence of leukemia [1]. It is known that children not achieving CR within four to six weeks of induction chemotherapy have the highest relapse rates [7]. In this study, all the cases were in morphological remission, with blast percentage on bone marrow ranging from 0% to 4% (<5%), and thus it did not correlate with MRD status. This suggests that all of them were in morphological/hematological remission; however, despite this, six were MRD-positive by MFC.

MRD detection by flow cytometry using the standard tube panel

Many studies have concluded that MRD negative or levels below 0.0001% at the end of induction therapy are associated with the best prognosis. Moreover, MRD levels above 0.01% at day 29 of induction are universally accepted to be associated with the highest risk of relapse [8-11]. This cut-off has been incorporated into many of the current management protocols, and trials are underway for assessing deeper levels of MRD.

In this study, MRD detection was done post-completion of induction chemotherapy. Around 24% were found to be MRD-positive, with the cut-off taken as >0.01% by the standard tube panel, which was taken as a gold standard. CD10 and CD20 were the only markers that were significantly different. The additional markers studied were CD 200, CD58, and CD38. When equal variance was not assumed between the two groups, CD200 emerged as a statistically significant marker. Interestingly, CD58 also seemed to be helpful as it showed a statistically significant trend, and more cases studied would identify its diagnostic usefulness. Coustan-Smith et al. also reported similar results but used a more extensive panel. They studied 200 pediatric B-ALL cases to identify novel markers for MRD detection. They worked with 30 markers, including CD10, CD19, CD34, CD58, CD200, CD123, and CD86. The results obtained by MFC were also verified using molecular techniques. They concluded that CD58 was one of the most useful markers as it was most differentially expressed in ALL cells, which was also confirmed by molecular studies. They also commented on the association of markers such as CD200, CD86, CD123, and CD97 with the presence of genetic abnormalities and thus correlated them with MRD detection and prognosis [12].

CD38, which was a marker of interest here, was studied in the MRD “lite” panel as well as the standard tube panel. It did not show any significant difference between the MRD-positive and MRD-negative cases. In a study on 304 AML and 138 ALL patients by Jiang et al., chromosome-positive ALL patients showed lower expression of CD38. They concluded in their study that higher expression of CD38 may be associated with a favorable outcome [13]. Hence, in our study, CD10, CD20, CD200, and CD58 were found valuable in MRD detection, which was concordant with many studies, including ours for CD200 and CD58 [14].

Standard tube panel versus MRD “lite” panel

MRD “lite” panel included CD38, CD10, CD19, CD34, and CD45 in our study, and the cut-off for positivity was taken as >0.01% of mononuclear events [15,16]. MRD was found positive in 36% (9/25) cases by the MRD “lite” panel. The percentage of MRD positivity ranged from 0.03% to 64.9%. All the cases showed a defined cluster of MRD-positive events in at least one of the marker combination plots. Positivity was calculated based on the plot showing the maximum number of events. Two observers analyzed all the cases to rule out observer bias. There was 100% concordance between the two observers, thus suggesting that the simplicity of the MRD “lite” panel reduces inter-observer variability. This supports our premise that it can be used with lesser expertise and hence has wider applicability as specialized centers are not widespread.

The sensitivity of the MRD “lite” panel taking the three-tube panel as the gold standard was 100%, and the specificity was 84.2% (Tables 6, 7). This implies that this panel will have no false-negative cases and can be used as an initial testing panel. For cases in which the outcome is not clear or there is some discrepancy in the results, a three-tube panel can be put in additionally, thereby making MRD detection easier and less expensive. Shaver et al. studied B-ALL MRD retrospectively, in which MRD was detected using a three-tube seven-color panel, and they developed an MRD “lite” eight-color panel, which they tested prospectively. They concluded that their optimized panel using CD9, CD10, CD19, CD20, CD34, CD38, CD45, and CD58 resulted in more efficient detection of MRD. They also found that the marker combination of CD38 and CD58 had the highest diagnostic efficacy [17]. In our study, this combination could not be studied as CD38 and CD58 were in separate tubes.

In another study conducted by Coustan-Smith et al., a simple and inexpensive MRD “lite” panel was made consisting of CD19, CD45, and CD10. Out of the 380 cases of B-ALL they studied, 55.5% showed MRD positivity, which was also confirmed by the complex MFC techniques and molecular tests [18]. Another study conducted in India by Chatterjee et al. included 15 cases of B-ALL for MRD analysis, which was performed using the MRD “lite” panel. This panel consisted of CD19, CD10, and CD34. They calculated MRD percentage positivity by measuring the co-expression of CD10 and/or CD34 in CD19-positive cells. Their study concluded that their panel could not differentiate between normal hematogones from blasts at the time of remission-induction [3]. In our study, we performed sequential gating and reverse gating to delineate the hematogone population and eliminate the blast population from this subset in different tubes. However, we also faced problems in separating hematogones by the MRD “lite” panel, which also led to false-positive results. Thus, the use of a more elaborate and comprehensive panel whenever resources permit is suggested. However, in both these studies, the MRD was performed on day 19, which is not the standard time for doing MRD in the currently used treatment protocols.

To conclude in our study, the MRD “lite” panel was as sensitive as the gold standard in detecting MRD positivity, suggesting that it is a good screening tool for detecting MRD. However, it was less specific as there were three false-positive cases, and more sensitive as there were no false-negative cases. Moreover, the MRD “lite” panel had 100% concordance between the two observers, suggesting simplicity in assessment and more objective evaluation, thus making it widely applicable. Further CD200 could be incorporated and tested for its use in the MRD “lite” panel.

## Conclusions

By embracing the MRD “lite” panel, we are making B-ALL MRD detection a more uncomplicated process for the technical team to reduce stain-to-gate time and for evaluators. However, we need to carefully design an algorithmic approach with an initial MRD “lite” panel having a very high sensitivity to minimize the chance of missing difficult cases in a fashion that is resource- and time-efficient. The conclusions reached in our study are based on a few cases and need to be validated on a larger number of cases using molecular techniques. Also, the outcome of these cases, if analyzed in future studies, could give valuable prognostic information. Studying the baseline expression with the expression pattern after induction chemotherapy could add to the specificity of the test, which could not be done in our study. Therefore, it is recommended that more studies be conducted to validate the MRD “lite” panel for its utility in detecting MRD.
